# Non-Invasive Spectroscopic Determination of the Skin and Blood Carotenoids of Term and Preterm Infants in the First Month of Life and the Influence of Free Radical-Mediated Diseases

**DOI:** 10.3390/life15040534

**Published:** 2025-03-24

**Authors:** Hanne Lademann, Maxim E. Darvin, Anna Häfke, Jürgen Lademann, Laura Wagner, Jan Däbritz, Dirk M. Olbertz

**Affiliations:** 1 Department of Pediatrics, Rostock University Medical Center, 18057 Rostock, Germany; haef_a@outlook.de; 2 Independent Researcher, 10178 Berlin, Germany; 3 Center of Experimental and Applied Cutaneous Physiology, Department of Dermatology, Venereology and Allergology, Charité—Universitätsmedizin Berlin, Corporate Member of Freie Universität Berlin and Humboldt-Universität zu Berlin, 10117 Berlin, Germany; juergenlademann@web.de; 4 Department of Pediatrics, University Medical School, 18057 Rostock, Germany; laura.wagner3@uni-rostock.de; 5 Department of Pediatrics, Greifswald University Medical Center, 17475 Greifswald, Germany; jan.daebritz@klinikumwb.de; 6 Department of Pediatrics, Klinikum Westbrandenburg, 14467 Potsdam, Germany; 7 Faculty of Medicine, HMU Health and Medical University, 14471 Potsdam, Germany; 8 Department of Neonatology, Südstadt Hospital Rostock, 18059 Rostock, Germany; dirk.olbertz@kliniksued-rostock.de

**Keywords:** oxidative stress, in vivo, antioxidants, free radicals, skin, stratum corneum, cutaneous carotenoids, newborn, skin scanner, reflectance spectroscopy

## Abstract

Postpartum adaptation causes an increased formation of free radicals (FRs) in the organism, which can lead to development of various FR-mediated diseases (FRMDs) in the newborn. The present study investigates the kinetics of skin and blood carotenoid antioxidants in term and preterm infants and the influence of FRMD. In the first phase, a diffuse reflectance spectroscopy-based scanner was validated for non-invasive measurements of skin carotenoids in term infants (thenar eminence) by correlation with blood carotenoids via reflection spectroscopy. In the second phase, the skin and blood carotenoids of 22 term and 13 preterm infants with and without FRMD were assessed from birth until discharge. It could be shown that the scanner reliably assessed carotenoids in the infants’ skin. The term and preterm infants showed similar kinetics of skin carotenoids, which increased and entered a plateau after 3–4 days. In our cohort, FRMD did not have a significant influence on skin carotenoid concentration. This was due to immature sweat glands and an insufficient excretion of carotenoids. Skin carotenoids seem to be unavailable, suggesting that they may have to be supplemented in infants with FRMD. Blood carotenoid concentrations tended to be lower in preterm infants and infants with FRMD compared to healthy term infants.

## 1. Introduction

Free radicals (FRs) are formed in the body during metabolism and by all forms of stress, such as environmental stress, diseases or nutritional disorders [1]. High concentration of FRs can result in development of oxidative stress, which lead to damage at the cellular and genomic level [2]. Newborns, especially preterm ones, are more susceptible to oxidative stress than term and later-in-life infants [3,4,5]. The human body has a protective system against the development of oxidative stress in the form of various antioxidants [1,6,7]. Firstly, there are antioxidants preventing the formation of FRs, such as albumin, transferrin and caeruloplasmin [8]. Secondly, so-called “chain-breaking” endogenous and exogenous antioxidants neutralize generated FRs, e.g., plant flavonoids, uric acid, vitamins A, C, E, carotenoids [8,9,10,11] and various enzymes [1,12,13,14].

During pregnancy, maternal placental transfer is the only source of antioxidants for the fetus. Studies have shown that fat-soluble vitamin E (tocopherol) [15,16,17,18] and carotenoids [19,20] are continuously transferred to the child via the placenta. The strong correlation between carotenoid concentration in maternal and infant cord plasma has been reported, with higher concentrations in maternal plasma [21,22]. Among blood carotenoids, ß-carotene, lutein and lycopene tend to be the most abundant [23]. It is also known that a high concentration of antioxidants can be found in early breast milk (colostrum) and serve as a protective source against the development of oxidative stress for the newborn [19,24,25,26]. However, due to the increased metabolism during pregnancy, FRs are continuously produced in the bodies of infants and their antioxidants are depleted [27,28,29]. Birth and adaptation to the environment also create oxidative stress in infants [30,31,32]. An enormous formation of FRs has been associated with the increased development of diseases in newborns [33]. These diseases were first summarized by Saugstad in 1988 under the term “oxygen radical diseases of the newborn” [34], later by Warner and Wispe in 1992 as “FR-mediated diseases” (FRMDs) [35] and specified by Perrone et al. as “FR-related diseases” [36]. The latter group also includes the main causes of mortality in the first 28 days of life, such as infections, complications of prematurity and asphyxia. These account for over 80% of neonatal mortality, or around 3.6 million cases per year worldwide [37], which highlights the importance of early diagnostics in newborns. A total of 25 to 30% of very-low-birth-weight infants suffer from late-onset sepsis (LOS), and the incidence of LOS in late preterm (34–37 weeks) infants is 6 to 10% [38]. In preterm infants in general, ≈26% of deaths occur due to LOS [39]. The mortality rate for newborns with sepsis is between 30 and 50% [40]. Other FR-related complications of preterm birth include retinopathy of prematurity [41], bronchopulmonary dysplasia [30], necrotizing enterocolitis [42] and hypoxic–ischemic encephalopathy [43]. It has been concluded that the determination of antioxidants in blood of preterm infants may be useful in the prediction of FR-related pathologies [44].

Antioxidants have multiple important roles throughout the human body [1,45]. The skin acts as a long-term reservoir for antioxidants [46], from which they can be released into the blood or absorbed from the blood as needed, and requires antioxidants as the main defense mechanism acting against FRs and the development of oxidative stress [47]. The antioxidant status of the blood, on the other hand, is subject to a different dynamic. Blood antioxidants are seen more as a short-term indicator [48] since they are consumed to neutralize acutely occurring FRs [1] and thus probably do not build up a long-term store. A proof for this was provided by Meinke et al. in 2010 [46]: After oral application of carotenoids, the blood and skin carotenoid concentrations were significantly increased. In the skin, however, it increased only with a delay and decreased later than in the blood. In addition, using electron paramagnetic resonance spectroscopy, it was shown that fat-soluble carotenoids can be considered as marker substances of the entire antioxidant status of the adult human epidermis in vivo [1,49].

In general, skin carotenoids can be measured invasively and non-invasively by various methods. The first possible method is an invasive measurement using high-performance liquid chromatography (HPLC) [50]. This method requires the collection of tissue samples or fluids from the body, such as skin biopsies and blood. In infants, for ethical reasons, only umbilical cord blood can be used as test material [36,51]. Umbilical cord blood reflects the intrauterine but not the extrauterine situation of the child and does not allow any conclusions regarding the concentration of antioxidants in the skin. Different biomarkers in the blood, such as total antioxidant capacity, glutathione reduced/oxidized ratio, malondialdehyde [52], 8-hidroxy-2′-deoxyguanosine [53] and others [4], can be used as diagnostic values to determine oxidative stress severity. It has already been shown that the invasive measurement of biomarkers of oxidative stress and FR-related damage (non-protein-bound iron, total hydroperoxides, advanced oxidation protein products) in cord blood can be an early identifier of preterm infants at risk of FRMD [54]. Kinetic studies, which require a larger number of samples, were not possible or only possible to a limited extent. In addition, antioxidants can be rapidly degraded by contact with oxygen, so the analysis of a skin biopsy may not prove informative. Thus, no assumption could be made on the kinetics of skin carotenoids. The second possible method of measuring skin carotenoids is their non-invasive determination with the help of optical technologies like, for example, resonance Raman spectroscopy (RRS), reflection spectroscopy or skin color measurements [55,56,57,58], with RRS being the current gold standard among non-invasive methods [59,60]. An RRS device is expensive, large, reacts very sensitively to external vibrations or shocks [59] and portable only to a limited extent; these are its main limitations. Thus, using RRS in delivery or in neonatal care units is complicated. The limited applicability of the previous methods for measuring skin carotenoid concentration in newborns (HPLC, RRS) poses a potential problem. To overcome these limitations, a miniature carotenoid skin scanner based on the multiple spatially resolved reflection spectroscopy (MSRRS) method, which shows a high correlation with RRS [61], could potentially be used in non-invasive infant skin research.

In 2013, Ermakov et al. [62] developed a variant of an RRS-based sensor specially adapted to newborns and found out that, compared to adults (*n* = 57), the average carotenoid concentration of their skin was significantly lower in infants aged 0 to 6 years (*n* = 32). In contrast to this, it was previously shown by Lademann et al. [29] that the skin carotenoid concentration of healthy term infants had significantly higher values than their mothers (*n* = 18), measured using a reflection spectroscopy-based miniaturized skin scanner and validated exclusively in adults [59]. A subgroup analysis of infants showed that newborns whose mothers suffered from pre-existing conditions or had pathologic weight gain during pregnancy had significantly lower carotenoid concentrations in the skin.

In accordance with the revised recommendations for appropriate weight gain during pregnancy published by the U.S. Institute of Medicine in 2009 [63], the data presented below in Table 1, which define pathologic weight gain in relation to pre-pregnancy body mass index, should be considered. Excessive weight gain during pregnancy has been shown to have a negative impact on the risk of developing gestational diabetes [64,65,66] and hypertensive disorders of pregnancy [67,68], as well as on the birth weight of the child [69] and the development of infants at high gestational ages [70]. This in turn may affect the mode of delivery, the necessity of labor induction [69] and potential obstetric complications due to macrosomia, such as shoulder dystocia, labor arrest or the need for a secondary cesarean section [69], to name just a few examples.

Carotenoid-containing-formula-fed preterm infants (*n* = 92) have an increased carotenoid concentration in their blood, which may suppress inflammation [71]. Furthermore, breast milk-fed preterm infants seem to have higher serum and skin carotenoid concentrations than formula-fed infants, suggesting that formula-fed infants may benefit from carotenoid supplementation (*n* = 40, 32 of whom were on breast milk) [72]. Thus, carotenoids significantly increased in the skin and serum of infants in the carotenoid-supplemented group compared to the control group [73], and their concentrations correlated slightly with the sensitivity of vitamin A deficiency [74].

Nevertheless, diode-based skin measurements have not been validated in newborn infants and no measurements have been performed in either premature infants or newborn infants with FR-related complications.

Thus, the aim of this study was to apply and investigate a non-invasive, diode-based carotenoid skin scanner firstly in the skin of term infants in comparison with validated, optical blood carotenoid measurements. Secondly, we exploratorily investigated the kinetics of carotenoids in the blood and skin of preterm infants, as well as the influence of FRMDs (hyperbilirubinemia, respiratory distress, infection). For this purpose, a further-developed carotenoid skin scanner was used, which works with the support of MSRRS, i.e., a new concept based on diffuse reflectance spectroscopy [61].

## 2. Materials and Methods

### 2.1. Patients

**Figure 1:**
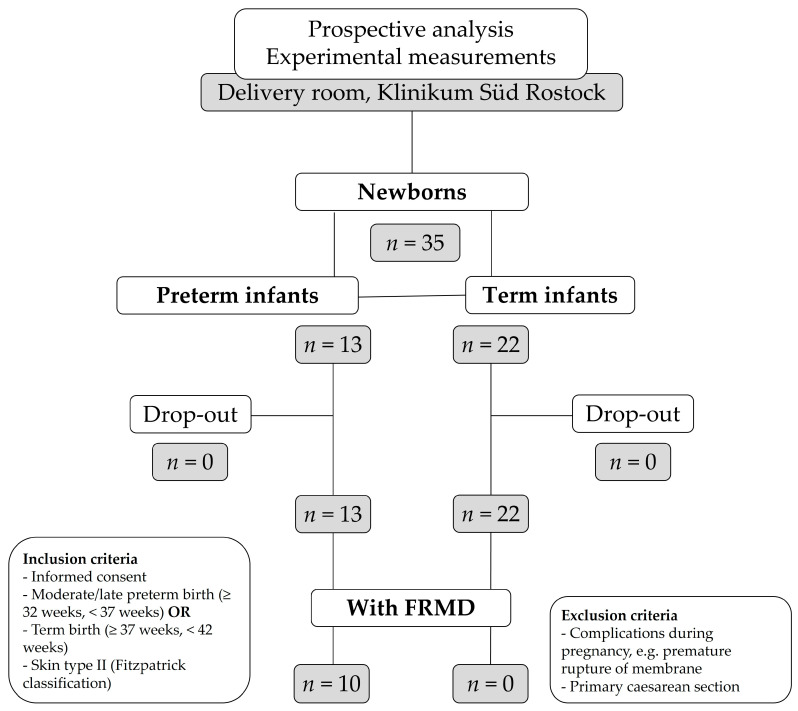
An overview of the study design and therapeutic groups. A total of 35 infants met the inclusion criteria, including 13 preterm and 22 term infants; 10 infants showed clinical signs of FRMD. Abbreviations: FRMD—free radical-mediated disease; *n*—number of subjects.

Forty pregnant women without any pathological symptoms, who had voluntarily registered for childbirth in the delivery room of Klinikum Südstadt Rostock, were informed about the study prior to/during labor or directly after birth. A total of 35 women gave their informed consent that their infants could participate in the study; 22 term and 13 preterm infants were investigated and none of them dropped out during the study period (Figure 1). Prior to enrolment, the following criteria were stipulated for inclusion in the study: informed written consent for participating in the study, moderate to late preterm (≥32 weeks, <37 weeks) and term infants (≥37 weeks, <42 weeks) and skin type II according to the Fitzpatrick classification [75]. The criteria for exclusion from the study were as follows: complications during pregnancy like the preterm rupture of the membrane and primary caesarean section. Since these are associated with an increased formation of FRs, the skin carotenoid concentration is thus lowered [29,76].

### 2.2. Ethics

The study was approved by the Ethics Committee of the Medical Faculty of the University of Rostock, Germany (Approval No.: A 2012-0070, 1 April 2015). All parents gave their written informed consent to participate in the study.

### 2.3. Study Protocol

The measurements were conducted as an observational study between gestational weeks 32+0 and 41+6 until discharge, depending on the gestational age at birth. Hence, the number of patients per gestational week varied. Skin carotenoids were measured non-invasively with MSRRS on the day of birth (day 0) and then on days 1, 2, 3, 4, 5, 6, 7, 10, 13, 17–24 and 27; blood carotenoids were determined on days 0, 1, 2, 3 and 4 after birth (Table 2).

Carotenoid concentrations were measured in the skin and blood of all the participating newborns. In addition to these measurements, a questionnaire was used to record both the child’s baseline characteristics (gender, somatic data, birth data: umbilical arterial pH, base deficit, APGAR score) and possible influencing factors (diet, nutritional supplement pre- and postnatal, clinical complications of the newborn (hyperbilirubinemia, respiratory distress, infection), maternal weight gain during pregnancy and pre-existing conditions).

### 2.4. Validation Experiments

#### 2.4.1. Skin Measurement

Little is known about kinetics of skin carotenoids in preterm infants so far; therefore, in this exploratory study daily skin measurements during the first week and two measurements from the second week onward were performed (Table 2).

**Figure 2:**
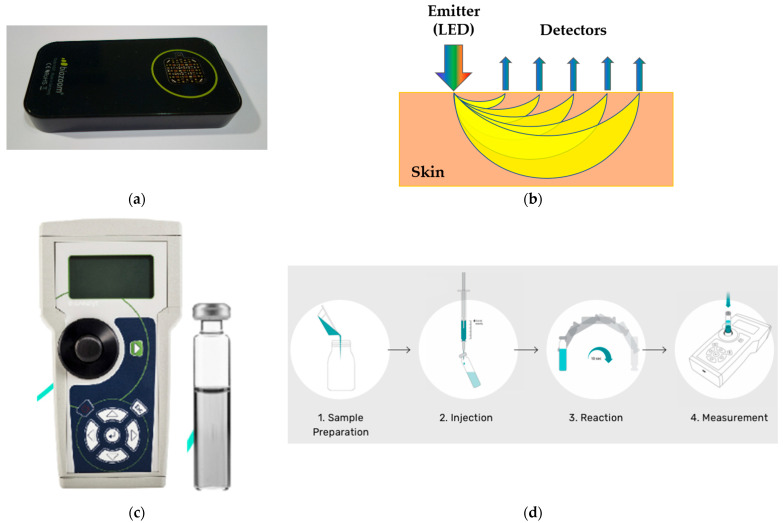
A representation of the devices used for the measurement of carotenoid concentration in the skin [top] and blood [bottom]: (**a**) Biozoom skin sensor to capture the cutaneous carotenoids at the ball of the thumb, functioning according to the principle of MSRRS [61]; (**b**) a schematic illustration of the MSRRS principle (1 emitting LED, 5 detectors); (**c**) an iCheck Carotenoid set for the collection of serum values; (**d**) the work steps for handling the iCheck Carotenoid set [77].

The carotenoid skin scanner was placed on the ball of the thumb. The measurement time was around 10 s. The skin scanner works according to the MSRRS method. This way, the portable skin scanner (biozoom, Kassel, Germany; Figure 2a) enables the non-invasive in vivo measurement of skin carotenoids. The sensor emits light via 118 different LED light sources which work in a spectral range between 350 and 1000 nm within and beyond the absorption maximum of the carotenoids (440–490 nm). The emitters and detectors of the scanner are in different cavities, causing the light to have to pass through the different skin depths before detection (Figure 2b). The light attenuated by the skin is collected by 152 detectors with filters for different spectral ranges, achieving an increased spectral resolution. The intensity and spectrum of the detected light depends on the specific absorption and scattering of the biomarkers located in the skin [61]. A mathematical algorithm was then applied to determine the concentration of skin carotenoids from the detected light. The measurement result of the skin scanner was represented on a scale with relative values between 0 and 10 arb. units. The measuring area of the thenar eminence was 4 cm^2^, which was specified as optimal by the manufacturer of the scanner in order to ensure better reproducibility of the data (for measurements on the thenar eminence of adults: measurement stability <10% within the total temperature range from 5 °C to + 30 °C and pressure contact between the skin and the MSRRS sensor from 800 Pa to 18,000 Pa [61]). An external validation on adult thenar skin showed a strong correlation of the total carotenoid concentrations between MSRRS and the previous “gold standard” of the non-invasive techniques—RRS—with *r* = 0.83. The same study calibrated the skin scanner to the level of serum carotenoids and thus achieved a correlation of *r* = 0.79 between blood and skin values [61].

The qualitative data measured by the MSRRS-based carotenoid skin scanner are valid for adult volunteers of skin types I–VI [61], as the measurements are taken at the palm of the hand where the melanin and blood chromophore concentrations are strongly reduced compared to other sites of the body [59].

#### 2.4.2. Blood Measurement

In addition, carotenoids were determined in the cord blood and in the context of the metabolic screening on the third day after birth. No additional blood samples but 0.2 mL more blood had to be taken. Carotenoids were recorded in the blood up to day four after birth. Measurements were conducted using a portable photometer (iCheck™, BioAnalyt GmbH, Germany; Figure 2c,d), which calculated the total carotene concentrations (absorption maximums 450 and 525 nm) in mg/L by measuring the color reaction of the blood serum samples. Carotene concentrations > 0.15 mg/L were detected with this fast and lab-independent technology. According to the manufacturer, the method is comparable to the two spectrophotometric reference methods, the AOAC (Association of Official Analytical Chemists) and HPLC [77]. There is a high correlation between the iCheck and AOAC (*r*^2^ = 0.99; *p* < 0.001) values, as well as between iCheck and HPLC (*r*^2^ = 0.94; *p* < 0.001) [78].

### 2.5. Statistics

Statistical analyses were performed by using IBM SPSS Statistics Version 27.0^®^ (IBM Corp. Released 2020. Armonk, NY, USA).

To describe metric variables, for example, birth weight and head circumference, mean value and standard deviation (SD) were used for all items. In addition, other measures of position and scattering such as minimum, maximum and quartile ranges were determined. Absolute and relative frequencies were collected for qualitative characteristics such as pediatric and birth history.

For the validation of the skin scanner, Spearman’s correlation analysis as a test for the nonlinear relationship between blood and skin carotenoids in term infants was performed.

Shapiro–Wilk tests and quantile–quantile plots were performed to detect a normal distribution (which was mainly not confirmed) of our cohort regarding baseline characteristics. A normal distribution was only confirmed for a few individual measurements; therefore, a non-parametric test method was applied due to the predominant absence of a normal distribution. To compare quantitative variables, the Mann–Whitney U test as a non-parametric test was used, as this is the test of choice for the comparison (testing for positional difference) of two independent and small samples with non-normally distributed populations. To reduce the alpha error, the Bonferroni correction was used to adjust for multiple testing.

The Fisher exact test was used to compare the nominal hospitalization data between preterm and term infants and between infants with and without FRMD. *p*-values < 0.05 were considered to be significant. Tables and line graphs are used for the exploratory presentation of significant results.

## 3. Results

An overview of the baseline characteristics of the cohort is shown in Table 3. Somatic parameters differ significantly between term and preterm infants, who were (as expected) younger, lighter and smaller (*p* < 0.001). Mothers of term newborns substituted vitamins prenatally (*n* = 19 vs. *n* = 6, *p* > 0.05) more often, while significantly more preterm than term infants received supplements (*n* = 4 vs. *n* = 0, *p* < 0.05) and iron (*n* = 6 vs. *n* = 0, *p* < 0.01). Regarding diet, significantly more preterm than term infants were fed with formula milk (*n* = 11 vs. *n* = 6, *p* < 0.01). FRMD only occurred in preterm infants (*n* = 10/13, 77%).

While maternal health status before pregnancy showed no difference, significantly more mothers of term infants suffered from pathological weight gain (*n* = 13 vs. *n* = 2, *p* < 0.05) (Table 3).

### 3.1. Validation

**Figure 3:**
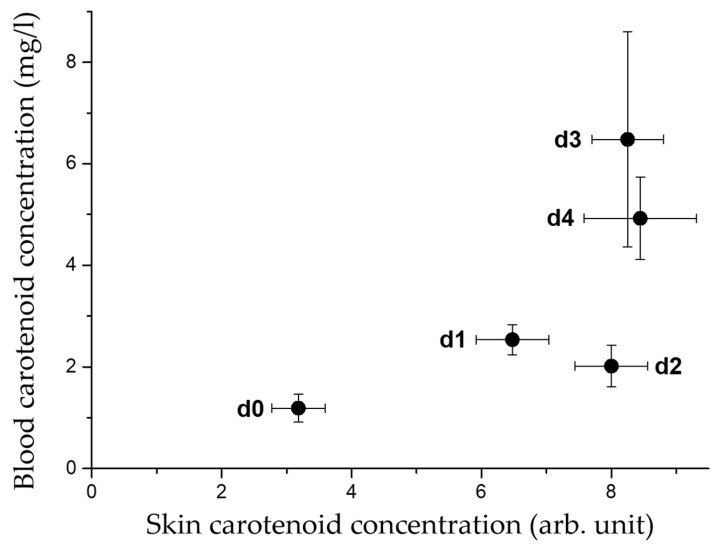
Mean ± standard error values per day of skin and blood carotenoid concentrations of 22 term infants from 5 measurements between the day of birth (d0) up to day four (d4). Abbreviations: arb. unit—arbitrary unit; d—day postpartum.

Correlation analysis shows that the skin and blood carotenoid concentrations of 22 term infants correlate positively (*r* = 0.8), with *p* = 0.10 as an indication of a tendentially significant correlation (Figure 3).

### 3.2. Kinetics of Carotenoids in Skin and Blood of Preterm vs. Term Infants

**Figure 4:**
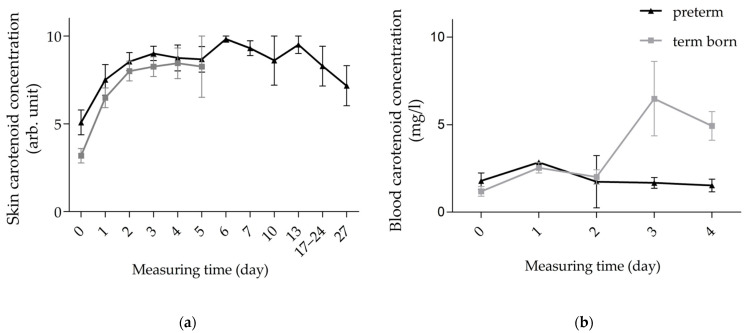
Kinetics of carotenoid concentrations in skin (**a**) and blood (**b**) of preterm (black, *n* = 13) and term infants (gray, *n* = 22). Abbreviations: arb. unit—arbitrary unit.

Our analysis of skin carotenoid concentration showed a value of 5.1 ± 2.5 arb. units already at the first measurement after delivery, observed in 13 preterm infants. During the first three days of life, the values increased to 9.0 ± 1.5 arb. units, maintained a plateau for the rest of the first and second week of life and then decreased towards the initial value on day 27 after delivery (7.17 ± 2.8 arb. units, *n* = 6; Figure 4a, Table S1). The values of the term infants measured during the first five days of life (Table 2) show comparable kinetics (Figure 4a). Blood measurements resulted in quite uniform carotenoid concentrations, with a value of 1.8 ± 1.7 mg/L after birth and 1.5 ± 0.7 mg/L on day four of life. Only on the first day after birth was the result slightly above the plateau, at 2.9 mg/L (Figure 4b, Table S1), but this was measured only in a single subject (Table 2).

Our analysis of gestational age as an influencing factor on carotenoid concentration showed no significant difference between preterm and term infants in both skin and blood. In skin, we only had comparable values up to day five postpartum. Up to this point, the kinetics of skin carotenoid concentration in the two groups were similar, tending to be slightly higher in the preterm than in the term infants. Both groups show an increase in skin carotenoid concentration up to day three postpartum, to 9.0 ± 1.5 arb. units in preterm vs. 8.3 ± 2.2 arb. units in term infants, transitioning to a plateau (Figure 4a). Carotenoid concentration in the blood of preterm infants remains at a low concentration plateau, while it reaches its maximum at 6.5 ± 4.2 mg/L on the third day postpartum and decreases again on the fourth day postpartum in term infants, remaining higher than in preterm infants (Figure 4b).

### 3.3. Kinetics of Carotenoids in Skin and Blood of FRMD vs. Non-FRMD Infants

The study included 10 infants with and 25 without FRMD (Table 3). Of the infants with complications, two suffered from sepsis, nine from hyperbilirubinemia and three from respiratory adaptation disorder (two infants had all three complications). Subgroup analysis shows no significant difference in skin carotenoid concentrations.

**Figure 5:**
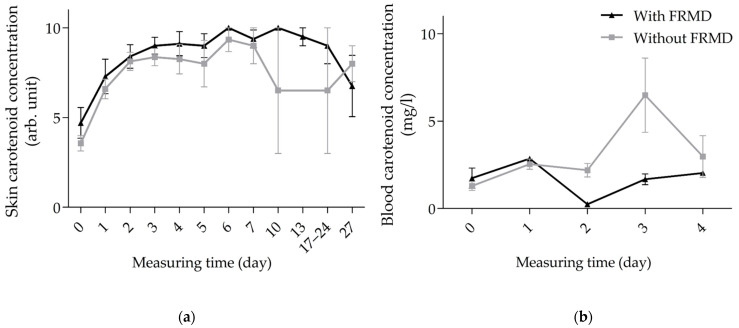
Kinetics of carotenoid concentration in skin (**a**) and blood (**b**) of infants with (black, *n* = 10) and without (gray, *n* = 25) FRMD. Abbreviations: arb. unit—arbitrary unit; FRMD—free radical-mediated disease.

High and stable skin carotenoid concentrations were detected in both groups, which decreased after the first week of life. The non-significant decline from day 10 postpartum was particularly evident in infants without FRMD, to 6.5 ± 5.0 vs. 10.0 ± 0.0 arb. units (Figure 5a, Table S2).

In blood, carotenoid concentrations tended to be lower in infants with FRMD than in healthy infants, but the difference was not significant (Figure 5b, Table S2).

## 4. Discussion

The aim of this study was firstly to investigate the effect of the skin carotenoid concentrations of term infants in comparison with a validated, optical measurement of carotenoids in the blood. Secondly, we exploratorily investigated the skin and blood carotenoid concentrations of preterm infants, including infants with developed FRMD (hyperbilirubinemia, respiratory distress, infection). Data could be collected from 22 term and 13 preterm infants at different time points after birth until the end of the first month of life. Regarding the baseline characteristics, there are significant differences between present groups, which could have an influence on the carotenoid concentration of the infants.

Here, vitamin supplementation prenatally in preterm infants is a positive influencing factor [79], whereas the administration of iron [30,32] and formula feeding [72], as well as the occurrence of FRMDs [34], are negative influencing factors. In term infants, pathological maternal weight gain certainly indicates as a negative influencing factor [29]. It should be noted that there are gaps in the demographic data, as individual parameters were not provided by mothers or infants (Table 3).

As part of the validation in infants, we compared the skin carotenoid concentration measured via an MSRRS-based carotenoid skin scanner [61] with blood carotenoid concentrations measured using the already validated iCheck method [77]. The correlation coefficient *r* = 0.8 shows a strong positive correlation with the blood reference method, which tends to be significant, with a *p* < 0.12. Spearman’s correlation analysis was performed, since the distribution of the measured values was nonlinear, and a normal distribution could not be assessed due to the small sample size. The accuracy of the measurements is reduced in preterm infants, who generally have very low carotenoid concentrations and thin, transparent skin, leading to large background absorption effects [62]. The obtained correlation agrees with results of Moran et al. [80], who investigated 4-month-old infants using reflectance spectroscopy (skin) and HPLC (blood). Nevertheless, the measurement of infant foreskin carotenoid concentration by RRS was valid compared to the HPLC method [72], underlining our results.

We also show that the kinetics of the skin carotenoids of preterm infants are comparable to term infants. This is surprising, as preterm infants with thinner skin [81,82] and lower weight have less subcutaneous fatty tissue, the reservoir for carotenoids and other fat-soluble antioxidants [48]. The development and maturation of organs in the fetus is largely completed by the 34th week of pregnancy. From this time onwards, it is mainly length growth that takes place. In addition, subcutaneous fatty tissue is then increasingly formed [83]. Throughout pregnancy, antioxidants are transferred from mother to the child via the placenta [15,16,17,18]. Since the preterm infants studied here showed a mean gestational age of 34 + 4 weeks of gestation, it seems that a reservoir had already been built up.

In addition to placental transmission, a high level of antioxidants is also transmitted to the child via the immature first breast milk, showing an increase up to day three after birth [24,25,26]. In our study, we were able to demonstrate a peak of carotenoid concentration in the skin of infants on the third day after birth, which is consistent with the results of previous observational studies [24,25,26]. The initial breast milk, called colostrum, is rich in antioxidants because these are already transported prenatally from the blood into the mammary glands—presumably to provide the newborn with a protective function against development of oxidative stress [24,25,26,84]. The maternal carotenoid concentration decreases equivalently in the corresponding time [29]. Such a behavior has already been observed in cows, as well [85,86]. In addition, as has been recently shown [87], carotenes and lycopene may be indispensable for the formation of the orthorhombic organization of lipids in the lamellae of the stratum corneum, which is directly related to the formation of skin barrier function in newborns.

Interestingly, FRMD showed no significant influence on the carotenoid concentration in skin and blood in this study (*n* = 10). Infants without complications (*n* = 25) show a stable plateau of carotenoid concentration with high values in both skin and blood. Blood analyses tend to show higher carotenoid concentrations in infants without FRMD, with a striking peak on day three postpartum. Therefore, preterm infants cannot utilize their reservoir of antioxidants in the skin in the case of complication development. This could be due to the fact that the release of antioxidants takes place partly via sweating [88], a process that is not yet possible to the full extent in preterm infants [89]. The positive influencing factors for antioxidant concentration mentioned above, especially postnatal vitamin administration, apparently compensate for this. Despite negative influences (iron, formula nutrition, FRMD), we found no significant differences in carotenoid concentration in the blood of term infants or preterm infants without FRMD.

To our knowledge, the present study is the first to non-invasively investigate the effect of FRMDs on the skin carotenoids of preterm infants. Nevertheless, this study shows some limitations. The method used for sensor validation is insufficient, as the Spearman analysis has low statistical power and the variables compared in the correlation are incorrectly treated as independent. Furthermore, the validity of the results is compromised by the lack of a statistically significant *p*-value, and the aforementioned skin conditions of preterm infants reduce the measurement accuracy.

Therefore, a more comprehensive study should be conducted in the future with a significantly larger cohort, including both term and preterm infants, incorporating invasive validation through HPLC methods in the skin. This study should provide a detailed assessment of the accuracy, precision and detection limits of the scanner across different skin types and physiological conditions. This was not possible in the non-invasive explorative study conducted for ethical reasons. It remains unclear to what extent seasons, times of day, and, for example, skin water content show an influence on skin carotenoids. In addition, this study was designed as an observational study, resulting in an inhomogeneous number of measurements on different days. It must be noted that the correlation analysis has limited test power with such a small number of subjects (*n* = 35) and such a small number of readings (*n* = 5); thus, existing differences may be missed. Furthermore, the analysis does not consider all the random effects. Thus, we anticipate that the small number of subjects is a major limitation of this study. However, the results are useful for testing hypotheses in large groups.

Future studies should investigate the kinetics of skin antioxidants (in particular, carotenoids) in preterm infants below 32 weeks of gestation. In addition, the relationship between the kinetics of known stress and infection parameters (cortisol, interleukin 6, C-reactive protein) and skin antioxidant concentration in term and preterm infants needs to be examined. In the future, such measurements could be used for diagnostics and monitoring in neonatology. Initial experimental studies in adults suggest that the severity of sepsis can be described by the presence of antioxidants, but also that outcomes can be improved by the administration of antioxidants [90,91,92,93].

## 5. Conclusions

This observational study on a small number of subjects (*n* = 35) shows that the diode-based optical skin scanner reliably assesses carotenoid concentration non-invasively in the skin of infants. Preterm and term infants with FRMD-related complications have lower blood carotenoid concentrations than preterm and term infants without postnatal complications. This could be because skin carotenoids are partly excreted via the sweat glands and absorbed into the skin via the blood, which is possible only to a limited extent in preterm and term infants. Consequently, skin carotenoid concentration remained high in both groups, suggesting that carotenoid antioxidants in human milk and/or supplements may compensate for the development of FRMD-related complications.

## Figures and Tables

**Table 1:** Recommendations for total weight gain during pregnancy, by pre-pregnancy BMI (pre-pregnancy BMI class based on World Health Organization definitions [63]). Abbreviation: BMI, body mass index.

Pre-Pregnancy BMI [kg/m^2^]	Pre-Pregnancy Class	Total Weight Gain Range [kg]
<18.5	underweight	12.5–18.0
18.5–24.9	normal weight	11.5–16.0
25.0–29.9	overweight	7.0–11.5
≥30.0	all classes of obesity	5.0–9.0

**Table 2:** Overview of timing of skin and blood measurements.

Measuring Time	Day After Birth	0	1	2	3	4	5	6	7	10	13	17–24	27
Skin													
Total	*n*	35	29	33	29	21	16	11	10	5	2	7	6
Preterm		13	8	13	13	12	12	11	10	5	2	7	6
Term		22	21	20	16	9	4	0	0	0	0	0	0
Blood													
Total	*n*	33	3	8	9	6							
Preterm		13	1	2	5	4							
Term		20	2	6	4	2							

Abbreviation: *n*—number of subjects.

**Table 3:** Baseline characteristics of term and preterm infants at birth/time of discharge.

		Term Infants	Preterm Infants		Missing Information
		*n* = 22	*n* = 13	*p*-Value	*n* (%)
female	*n* (%)	14 (63.6)	10 (76.9)	ns b	0
gestational age [weeks + days]	mean ± SD	40 + 1 ± 1 + 2	34 + 4 ± 1 + 5	*** a	0
birth weight [gram]		3544 ± 374	2178 ± 414	*** a	1 ti
head circumference [cm]		35.4 ± 1.6	31.4 ± 2.16	*** a	3 ti 3 pi
substitution of vitamins	*n* (%)				
pre-natal		19 (86.4)	6 (46.2)	ns b	2 ti 4 pi
post-natal		0 (0.0)	4 (30.8)	* b	1 ti
substitution of iron					
pre-natal		15 (68.2)	4 (30.7)	ns b	2 ti 4 pi
post-natal		0 (0.0)	6 (46.2)	** b	1 ti
diet					
human breast milk		19 (86.4)	12 (92.3)	ns b	0
formula milk		6 (27.3)	11 (84.6)	** b	0
pathological maternal weight gain during pregnancy		13 (59.1)	2 (15.4)	* b	0
maternal pre-existing conditions		10 (45.5)	3 (23.1)	ns b	0
FRMD		0	10 (76.9)	*** b	1 ti
hyperbilirubinemia		0 (0.0)	9 (69.2)	*** b	1 ti
respiratory distress		0 (0.0)	3 (23.1)	* b	1 ti
sepsis		0 (0.0)	2 (15.4)	ns b	1 ti

a—Mann–Whitney U test; b—Fisher’s exact test; ns (non-significant) *p* ≥ 0.05; * *p* < 0.05; ** *p* < 0.01; *** *p* < 0.001. Abbreviations: *n*—number of subjects; SD—standard deviation; FRMD—free radical-mediated disease; ti—term infants; pi—preterm infants.

## Data Availability

The raw data supporting the conclusions of this article will be made available by the authors, upon reasonable request.

## Supplementary Materials

The following supporting information can be downloaded at: https://www.mdpi.com/article/10.3390/life15040534/s1, Table S1: Skin and blood carotenoids of term and preterm infants.; Table S2: Skin and blood carotenoids of infants with and without FRMD.
