# Supplementary material for: Non-Invasive Spectroscopic Determination of the Skin and Blood Carotenoids of Term and Preterm Infants in the First Month of Life and the Influence of Free Radical-Mediated Diseases
Source: Life (Basel). 2025 Mar 24;15(4):534. doi: 10.3390/life15040534 (PMC12028751; doi:10.3390/life15040534)
Supplement: Supplementary file 1 [file life-15-00534-s001.zip › life-3386705-supplementary.pdf]

**Table S1.** Skin and blood carotenoids of term and preterm infants.

| | | **Term Infants** | **Preterm Infants** | |
|---|---|---|---|---|
| | ***n* (%)** | **22 (62.9)** | **13 (37.1)** | **p[a]** |
| **Skin carotenoids** | | | | |
| Birth | mean ± SD (*n*) | 3.2 ± 1.9 (22) | 5.1 ± 2.5 (13) | n.s. |
| d 1 | | 6.5 ± 2.6 (21) | 7.5 ± 2.4 (8) | |
| d 2 | | 8.0 ± 2.5 (20) | 8.5 ± 1.9 (13) | |
| d 3 | | 8.3 ± 2.2 (16) | 9.0 ± 1.5 (13) | |
| d 4 | | 8.4 ± 2.6 (9) | 8.8 ± 2.5 (12) | |
| d 5 | | 8.3 ± 3.5 (4) | 8.7 ± 2.5 (12) | |
| **Blood carotenoids** | | | | |
| Birth | mean ± SD (*n*) | 1.2 ± 1.2 (20) | 1.8 ± 1.7 (13) | |
| d 1 | | 2.5 ± 0.4 (2) | 2.9 (1) | |
| d 2 | | 2.0 ± 1.0 (6) | 1.7 ± 2.1 (2) | |
| d 3 | | 6.5 ± 4.2 (4) | 1.7 ± 0.7 (5) | |
| d 4 | | 4.9 ± 1.2 (2) | 1.5 ± 0.7 (4) | |

a – Mann Whitney-U-Test;
Abbreviations: *n* – number of subjects; SD – standard deviation; d – day; n.s. – non-significant.

**Table S2.** Skin and blood carotenoids of infants with and without FRMD.

| | | With FRMD | Without FRMD | |
|---|---|---|---|---|
| | *n* (%) | 10 (28.6) | 25 (71.4) | **p[a]** |
| **Skin carotenoids** | | | | |
| Birth | mean ± SD (*n*) | 4.7 ± 2.7 (10) | 3.6 ± 2.1 (25) | n.s. |
| d 1 | | 7.3 ± 2.6 (7) | 6.6 ± 2.6 (22) | |
| d 2 | | 8.4 ± 2.1 (10) | 8.1 ± 2.4 (23) | |
| d 3 | | 9.0 ± 1.5 (10) | 8.4 ± 2.1 (19) | |
| d 4 | | 9.1 ± 2.0 (9) | 8.3 ± 2.8 (12) | |
| d 5 | | 9.0 ± 2.0 (9) | 8.0 ± 3.4 (7) | |
| d 6 | | 10.0 ± 0.0 (8) | 9.3 ± 1.2 (3) | |
| d 7 | | 9.4 ± 1.4 (8) | 9.0 ± 1.4 (2) | |
| d 10 | | 10.0 ± 0.0 (3) | 6.5 ± 5.0 (2) | |
| d 17–24 | | 9.0 ± 2.2 (5) | 6.5 ± 5.0 (2) | |
| d 27 | | 6.8 ± 3.4 (4) | 8.0 ± 1.4 (2) | |
| **Blood carotenoids** | | | | |
| Birth | mean ± SD (*n*) | 1.7 ± 1.8 (10) | 1.3 ± 1.2 (23) | |
| d 1 | | 2.9 (1) | 2.5 ± 0.4 (2) | |
| d 2 | | 0.2 (1) | 2.2 ± 1.0 (7) | |
| d 3 | | 1.7 ± 0.7 (5) | 6.5 ± 4.2 (4) | |
| d 4 | | 2.0 ± 0.0 (2) | 3.0 ± 2.4 (4) | |

a – Mann Whitney-U-Test
Abbreviations: n – number of subjects; SD – standard deviation; d – day; FRMD – free radical-mediated diseases; n.s. – non-significant.
